# PAF49: An RNA Polymerase I subunit essential for rDNA transcription and stabilization of PAF53

**DOI:** 10.1016/j.jbc.2023.104951

**Published:** 2023-06-24

**Authors:** Rachel McNamar, Emma Freeman, Kairo N. Baylor, Aula M. Fakhouri, Sui Huang, Bruce A. Knutson, Lawrence I. Rothblum

**Affiliations:** 1 Department of Cell Biology, University of Oklahoma College of Medicine, Oklahoma City, Oklahoma, USA; 2 Department of Cell and Development Biology, Northwestern University Feinberg School of Medicine, Chicago, Illinois, USA; 3 Department of Biochemistry and Molecular Biology, SUNY Upstate Medical University, Syracuse, New York, USA

**Keywords:** RNA polymerase I (RNAP1), ribosomal RNA, auxin, nucleolus, PAF53, RNA polymerase I subunit E (POLR1E), PAF49, RNA polymerase I subunit G (POLR1G), conditional protein degradation, SCF^TIR1^ (TIR1), transcription

## Abstract

The application of genetic and biochemical techniques in yeast has informed our knowledge of transcription in mammalian cells. Such systems have allowed investigators to determine whether a gene was essential and to determine its function in rDNA transcription. However, there are significant differences in the nature of the transcription factors essential for transcription by Pol I in yeast and mammalian cells, and yeast RNA polymerase I contains 14 subunits while mammalian polymerase contains 13 subunits. We previously reported the adaptation of the auxin-dependent degron that enabled a combination of a "genetics-like" approach and biochemistry to study mammalian rDNA transcription. Using this system, we studied the mammalian orthologue of yeast RPA34.5, PAF49, and found that it is essential for rDNA transcription and cell division. The auxin-induced degradation of PAF49 induced nucleolar stress and the accumulation of P53. Interestingly, the auxin-induced degradation of AID-tagged PAF49 led to the degradation of its binding partner, PAF53, but not vice versa. A similar pattern of co-dependent expression was also found when we studied the non-essential, yeast orthologues. An analysis of the domains of PAF49 that are essential for rDNA transcription demonstrated a requirement for both the dimerization domain and an “arm” of PAF49 that interacts with PolR1B. Further, we demonstrate this interaction can be disrupted to inhibit Pol I transcription in normal and cancer cells which leads to the arrest of normal cells and cancer cell death. In summary, we have shown that both PAF53 and PAF49 are necessary for rDNA transcription and cell growth.

Ribosome biogenesis is an essential component of the homeostatic mechanisms for cell survival. A dividing cell must replicate its ribosomes every cell cycle. Further, cells that are hypertrophic must increase their ribosome content in order to accommodate the increased accumulation of protein (1, 2, 3, 4). Along these same lines, increased rates of ribosome biogenesis are a hallmark of cancer cells (5, 6). A mammalian cell contains approximately 4 × 10^6^ cytoplasmic ribosomes, which account for 80% of total cellular RNA and 5% to 10% of the cellular protein (7, 8, 9). The process of ribosome biogenesis includes the coordinated expression of approximately 80 ribosomal protein genes, 300 proteins and snoRNAs involved in pre-rRNA processing, and the 600 to 800 5SRNA and ∼150 to 200 active pre-rRNA genes (10). The formation of ribosomes is energy intensive and complex. Moreover, the process is cell type-specific and subject to regulation at multiple levels. The dysregulation of ribosome biogenesis is associated with cancer and a group of disorders referred to as ribosomopathies (5, 11, 12, 13, 14).

The rate-limiting step in ribosome biogenesis is the transcription of the pre-rRNA genes by RNA polymerase I (15, 16, 17). RNA polymerase I uses a unique set of general transcription factors. Interestingly, the yeast and mammalian transcription factors are structurally distinct from one another. The mammalian core Pol I transcription factors include UBF1 (18, 19) and SL1, comprised of TBP and four TATA-associated proteins specific for transcription by Pol I (20, 21, 22, 23). The yeast factors include TBP, core factor (Rrn7, Rrn6 and Rrn11), and upstream activating factor (Rrn9, Rrn5, Rrn10, Uaf30, and histones H3 and H4) (24, 25, 26, 27). Unlike their transcription factors, mammalian and yeast RNA polymerase I are structurally similar and contain nearly identical subunits.

A fully functional molecule of yeast RNA polymerase I consists of 15 subunits (28, 29). This total includes the core Pol I, a heterodimer of RPA49-RPA34.5 and RRN3. Five of the core subunits are shared with the other two polymerases and two are uniquely shared with Pol III. A fully functional molecule of mammalian Pol I consists of 14 subunits: 11 core Pol I subunits, and three **P**olymerase **A**ssociated **F**actors referred to as RRN3 and a heterodimer of PAF53-PAF49 (30). RRN3, PAF53, and PAF49 are the mammalian orthologues of yeast RRN3, and the heterodimer of RPA49 and RPA34.5, respectively. The role(s) of the heterodimer in rDNA transcription are still subject to investigation. Previous studies in yeast demonstrated that neither RPA49 nor RPA34.5 was essential for rDNA transcription and cell proliferation. For example, deletion of yeast RPA49 results in colonies that grow at 6% of the wild type rate at 25 °C (31), and deletion of the partner in the heterodimer, RPA34.5, has only a minor effect on growth or rRNA synthesis, but Pol I loses its RPA49 subunit upon purification (32).

Muramatsu’s laboratory reported that the Pol I subfraction that was active in rDNA transcription *in vitro* contained PAF53 and PAF49. Further, they found that PAF53 and PAF49 were essential for promoter-specific transcription (33, 34). Based on the observations that only 60% of Pol I complexes contained PAF53 (35) and that the association of the PAFs with core Pol I was regulated (36), we hypothesized this fraction defined transcription-competent Pol I and that these factors had essential roles in rDNA transcription. This was confirmed by recent genomic screens that identified PAF53 and PAF49 as being essential genes (37, 38, 39, 40).

While genetic studies in yeast and mammalian cells have provided evidence that these factors play a significant role in rDNA transcription, their specific roles are unknown. To investigate this question, we developed a system that would allow us to rapidly knock down the level of a targeted transcription factor and thus enable the study of its role in the biology and biochemistry of the cell. In a previous article, we reported on the construction of a cell line that expresses the *O. sativa* TIR1 linked to a nuclear localization signal (41). This enabled us to induce the rapid degradation of any protein linked to the auxin-inducible degron of IAA17 (AID). We then used CRISPR/Cas9 and homologous recombination to tag both PAF53 alleles in HEK293 cells. That study demonstrated that PAF53 was essential for rDNA transcription and cell proliferation. Further, we were able to identify an essential, novel DNA-binding domain in mammalian PAF53 which we subsequently identified in its yeast ortholog, RPA49.

We have now extended our studies to mammalian PAF49. We have found that the auxin-induced degradation of PAF49 inhibits rDNA transcription and cell division (42). Structural studies of yeast RPA34.5 demonstrated that the molecule contained an N-terminal dimerization domain and an “arm” that appears to mediate its interaction with the A135 subunit of Pol I (28, 29, 43). The remainder of the molecule is not found in cryo-EM studies. We have found that the dimerization domain of PAF49 is insufficient to rescue rDNA transcription and cell cycle progression. These activities required both the dimerization domain and the next 100 amino acids. Further, we found that the knockdown of PAF49 results in the relatively rapid degradation of PAF53 but not other subunits of Pol I. We subsequently found that a similar situation occurs in yeast; RPA49 accumulation is dependent on RPA34.5. We also observed that the rescue of PAF53 levels depends on that portion of PAF49 which was also required to rescue rDNA transcription. Based on these observations, we identified a conserved 25 amino acid domain in PAF49 and found that when that peptide was transduced into non-transformed HEK293 cells it inhibited cell proliferation.

## Results

**Figure 1:**
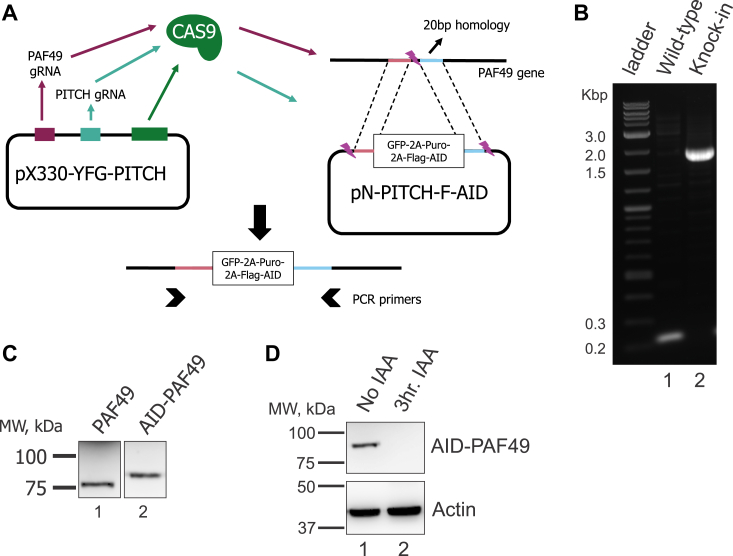
**Targeting PAF49 with an AID.***A*, schematic of the microhomology-mediated end joining (MMEJ) tagging technique used to knock-in (KI) an auxin-inducible degron (AID) at the N-terminus of the human PAF49 gene in HEK293 cells expressing TIR1 (97). The pX330 vector expresses Cas9 and two gRNAs, one targeting the first exon of the hPAF49 gene and the other targeting the pN-PITCh vector to release the repair fragment containing the AID tag. The pN-PITCh vector contains GFP, the puromycin resistance gene, and a FLAG-tagged AID sequence. All components are separated by self-cleaving T2A and P2A peptides. Two 20-bp microhomology regions corresponding to the regions directly adjacent to the Cas9 cleavage site are flanking the insertion cassette. The pN-PITCh and pX330 vectors are co-transfected, and cells are selected with puromycin and subject to dilution cloning. *B*, PCR analysis of HEK293 cells negative and positive for homozygous knock-in (KI). Primers that flanked the insert were used to ensure KI occurred at the desired gene locus. Cells were negative if PCR produced a product of 250 bp while PCR analysis of positive cells produced a product of approximately 1,900 bp. *C*, examples of untagged and tagged hPAF49 in HEK293 cells as determined by Western blot analysis with anti-PAF49 antibody. *D*, treatment with 1 mM indole-3 acetic acid (IAA) results in the degradation of AID-PAF49 in 3 h.

CRISPR-driven microhomology-mediated end joining (MMEJ) was utilized to tag both alleles of the endogenous PAF49 gene with an auxin-inducible degron (AID) at the N-terminus (Fig. 1*A*) in HEK293 cells that constitutively expressed *O. sativa* TIR1 as previously describe (41, 44, 45). The clones were screened by genomic PCR to confirm the presence of the recombinant insert (Fig. 1*B*). Clones that were negative would show a PCR product of 250 bp (Fig. 1*B*, *lane 1*) while clones that were positive for the insert would produce a PCR product of approximately 1900 bp (Fig. 1*B*, *lane 2*). The genomic PCR was also used to select clones that had both alleles tagged. The PCR products were cloned, and four clones were sequenced. The sequences were identical and did not demonstrate errors in the recombination of the inserted sequence (data not shown).

After the genomic PCR screen, positive clones were treated with or without 1 mM indole-3-acetic acid (IAA) for 3 h and harvested for Western blot analysis. Figure 1*C* shows the difference in size between untagged and tagged hPAF49 (*lanes 1 and 2* respectively). As demonstrated in Figure 1*D*, AID-PAF49 is degraded rapidly in the presence of IAA. Our lab has found this system to have multiple advantages when compared to RNAi technology and CRISPR knock out (KO). For example, some proteins like PAF49 have a long half-life and take multiple days to be completely knocked down (46) by RNAi technologies such as siRNAs. The TIR1-AID system allows us to knock down our protein of interest within 3 h which prevents the cells from having time to compensate for the gradual loss of protein. Our lab has also shown that some genes, such as PAF53, are essential, and stable CRISPR KO lines cannot be selected (47). Again, the AID system circumvents that issue.

Previous studies performed in yeast have demonstrated that RPA34, the homolog of mammalian PAF49, is not essential for rDNA transcription by Pol I, cell proliferation, or cell viability (31, 32, 48, 49). On the other hand, multiple CRISPR screens performed in mammalian cells looking for genes “required for proliferation and survival” found that the PolR1G (PAF49) gene was essential (37, 38). Additionally, Muramatsu’s laboratory has demonstrated that the treatment of nuclear extracts with an anti-PAF49 antibody inhibited Pol I transcription (34). These studies highlight one of the multiple differences between the mammalian and yeast Pol I systems, signifying the importance of studying PAF49 and its role in Pol I transcription in mammalian cells. We therefore sought to use the AID system to knock down PAF49 in HEK293 cells in order to confirm the studies previously performed in mammalian cells that show PAF49 to be essential for Pol I transcription and cell proliferation/viability.

**Figure 2:**
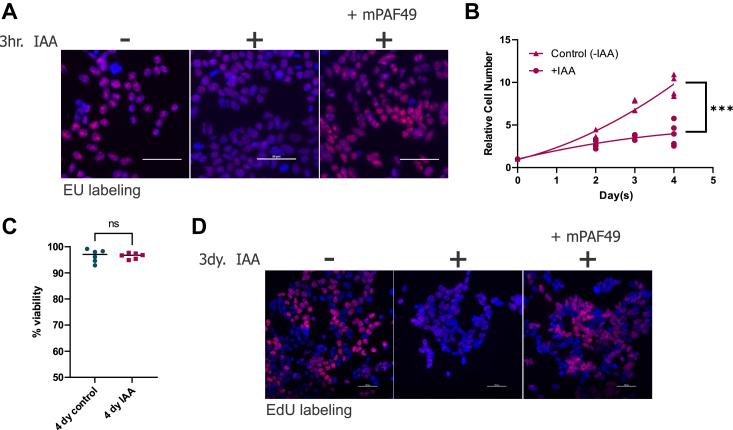
**Depletion of PAF49 results in the inhibition of rDNA transcription and cell proliferation.***A*, after cells were treated with 1 mM IAA for 3 h, they were pulsed with 5-ethynyl uridine (EU) for 15 min, and *de novo* synthesized RNA (*red*) was visualized as described against a DAPI background (94). To rescue rDNA transcription, wild-type mouse PAF49 (WT mPAF49) was ectopically expressed for 24 h before the cells were treated with IAA. Scale bar = 50 μm. *B*, cells containing AID-PAF49 were treated with 1 mM IAA or vehicle for 4 days. On days 0, 2, 3, and 4, live cells were counted as described (79). The growth curves were constructed with a nonlinear fit with GraphPad Prism. Significance between day 4 control and IAA data points was determined by a two-tail *t* test, *p* = 0.0003. *C*, trypan blue exclusion was used to count live cells and measure percentage viability within the cell population. A two-tail *t* test was performed to test for significance, *p* = 0.9762. *D*, after cells were treated with 1 mM IAA for 3 days, they were pulsed with ethynyl-2′-deoxyuridine (EdU) for 1 h, and *de novo* synthesized DNA (*red*) was visualized as described against a DAPI background (94). The ectopic expression of WT PAF49 rescued cell proliferation. WT mPAF49 was ectopically expressed for 24 h before the cells were treated with IAA. Scale bar = 100 μm.

As shown in Figure 2*A*, treatment with IAA for 3 h to knock down PAF49 resulted in the inhibition of rDNA transcription as demonstrated by EU labeling of newly synthesized RNA. This inhibition of rDNA transcription could be rescued with ectopically expressed wild-type mouse PAF49 (Fig. 2*A*). Previous studies have demonstrated that there is a connection between rDNA transcription and cell proliferation/viability. When any step in ribosome biogenesis is inhibited, a phenomenon referred to as “nucleolar stress” or “ribosome stress” occurs. Depending on the physiology of the cell, nucleolar stress can lead to cell cycle arrest and/or cell death (50, 51, 52, 53, 54, 55, 56, 57). Experiments performed in Figure 2, *B* and *C* show that PAF49 is essential for cell proliferation but not cell viability. Specifically, in comparison to untreated cells, there is a significant reduction in proliferation in cells treated with IAA after 4 days (Fig. 2*B*). Moreover, trypan blue exclusion demonstrated no significant difference in percentage viability (Fig. 2*C*). An alternative method to looking at whether the cells are actively progressing through the cell cycle is determining if they are replicating their DNA. When PAF49 was knocked down, EdU incorporation into newly synthesized DNA was inhibited (Fig. 2*D*). We were able to rescue DNA synthesis with the ectopic expression of WT mPAF49 (Fig. 2*D*). These results confirm the conclusions from previous studies that PAF49 is essential for rDNA transcription and cell proliferation. Alternatively, PAF49 is not required for cell viability. Additionally, we showed that the inhibition of rDNA transcription and cell cycle arrest were not due to treatment with IAA as we could rescue both rDNA transcription and DNA synthesis with ectopically expressed PAF49 in the presence of IAA.

**Figure 3:**
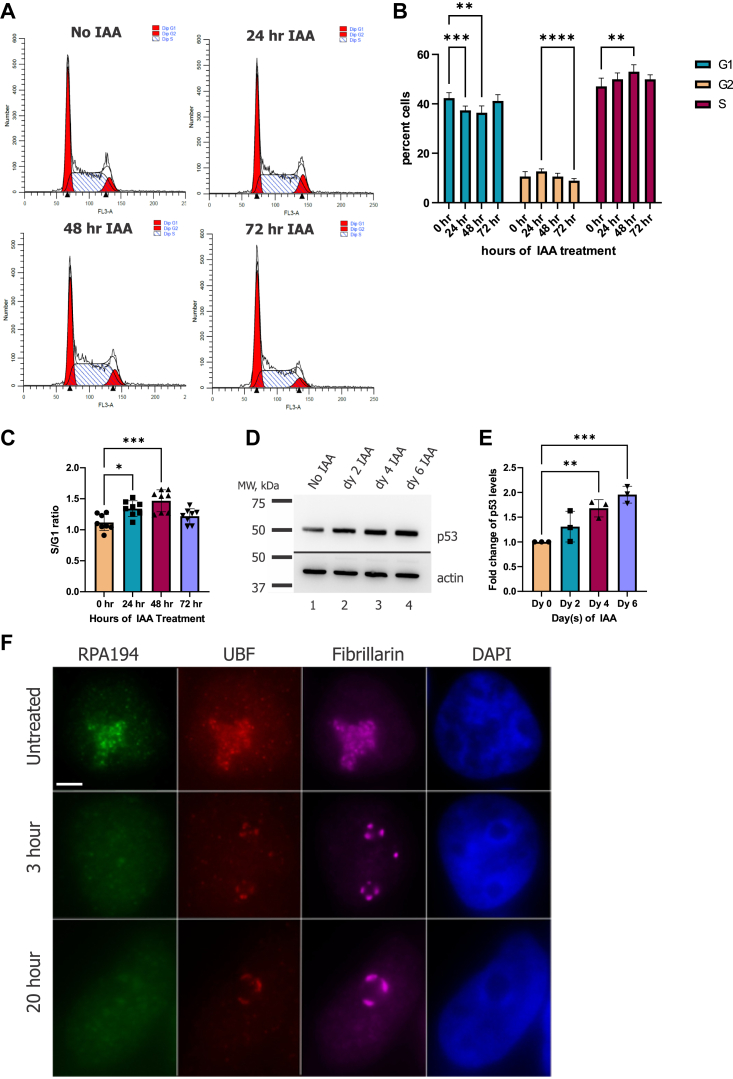
**Depletion of PAF49 does not cause cell cycle arrest in a specific phase.***A*, FACS analysis of cells at the indicated times post-treatment of IAA. *B*, quantification of the distribution of cells in G_1_, S, or G_2_ following IAA treatment. Four independent repeats of the analyses presented in (*A*) were each carried out in duplicate. The data were analyzed by two-way ANOVA with Tukey’s multiple comparisons test. *C*, ratio of cells in S and G_1_ was calculated for the data presented in (*B*). Significance was determined by a one-way ANOVA with Tukey’s multiple comparisons test. *D*, Cells containing AID-PAF49 were treated with 1 mM IAA or vehicle for up to 6 days. On days 0, 2, 4, and 6, cells were harvested and lysed in HEPES lysis buffer. The expression of p53 was determined by Western blot analysis with anti-p53 antibody. *E*, three independent repeats of the Western blot data presented in (*D*) were performed. The levels of p53 were corrected for the signal of the housekeeping protein β-actin. The data were analyzed by one-way ANOVA with Dunnett’s multiple comparisons test. The error bars represent mean ± SD; ∗ = 0.05 to 0.01, ∗∗ <0.01, ∗∗∗ <0.001, ∗∗∗∗ <0.0001. *F*, cells containing AID-PAF53 were treated with 500 μM IAA for 3 h and 20 h. Reduction of PAF53 shows reorganization of nucleolar structures into caps along the periphery, where UBF and fibrillarin colocalize and RPA194 disperses into the nucleoplasm. Scale bar = 5 μm.

To further understand how the cells were arresting when PAF49 was knocked down, we performed a FACS analysis of fixed cells stained with propidium iodide (Fig. 3, *A*–*C*). Cells were treated with IAA and then fixed after 0 h, 24 h, 48 h, and 72 h of treatment. In Figure 3*B*, we show that after 48 h of IAA treatment, there is a slight but significant decrease in cells in G_1_ and an increase in cells in the S phase. Further, there was a small but significant decrease in cells in G_2_ after 72 h of IAA treatment. Additionally, we see a significant increase in the S/G_1_ ratio after 48 h (Fig. 3*C*). The cell cycle distribution was consistent with the data we observed when we knocked down PAF53 with the same AID system (41).

Downstream cell cycle arrest due to nucleolar stress can be caused by p53-dependent and/or independent pathways (50, 51, 52, 56). Therefore, we determined if there was an increase in p53 levels when PAF49 was knocked down for 6 days. As illustrated in Figure 3 (panels D and E), Western blot analysis demonstrated that there was an increase in p53 protein levels consistent with the model that the cells arrested in a p53-dependent manner. Another hallmark of nucleolar stress is the reorganization of the nucleolus into a ring-like structure with caps (58, 59). When cells were treated with IAA, the nucleolus began to reorganize into a similar structure within 3 h (Fig. 3*F*). We observed UBF, a component of the Pol I pre-initiation complex, and fibrillarin, a constituent of the fibrillar and dense fibrillar components of the nucleolus involved in pre-rRNA methylation, localize into caps at the periphery of the nucleolus. Alternatively, RPA194, the highest molecular weight subunit of the Pol I holoenzyme, disperses into the nucleoplasm.

**Figure 4:**
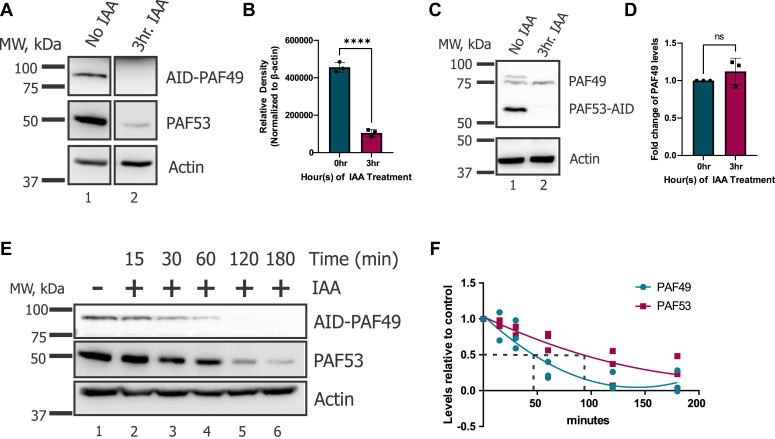
**Knockdown of PAF49 results in the rapid degradation of PAF53, but not vice versa.***A*, cells were treated with 1 mM IAA or vehicle for 3 h to knock down AID-PAF49 levels. Cells were then harvested and lysed in HEPES lysis buffer. The expression of AID-PAF49 and PAF53 was determined by Western blot analysis. *B*, three independent repeats of the Western blot experiment presented in *A* were performed. The levels of PAF53 were corrected for β-actin. The data were analyzed by a two-tail *t* test, *p* < 0.0001. *C*, cells with PAF53-AID were treated with 1 mM IAA or vehicle for 3 h and were harvested and lysed in HEPES lysis buffer. The expression of PAF53-AID and PAF49 was determined *via* Western blot analysis. *D*, three independent repeats of the Western blot experiment presented in *C* were performed. The levels of PAF49 were corrected with β-actin. A two-tail *t* test was performed to test for significance, *p* = 0.3007. *E*, cells expressing AID- PAF49 were treated with 1 mM IAA for 3 h and were harvested and lysed at the indicated time points: 0, 15, 30, 60, 120, and 180 min after IAA treatment. Western blot analysis was performed to determine the protein levels of AID-PAF49 and PAF53 at each time point. *F*, three independent repeats of the experiment presented in *E* were performed. The levels of AID-PAF49 and PAF53 were corrected for the β-actin content of the sample. The change in AID-PAF49 and PAF53 levels with time were plotted with a nonlinear fit with GraphPad Prism. The *dotted lines* indicate the approximate half-life of each protein in these experiments.

As previously mentioned, PAF49 heterodimerizes with PAF53 (28, 36, 60, 61). Therefore, we sought to determine whether the expression of PAF49 and PAF53 are dependent on one another. To test this, we quantified the levels of PAF53 when PAF49 was knocked down *via* Western blot analysis (Fig. 4, *A* and *B*). Figure 4*B* shows a significant decrease in PAF53 levels. Alternatively, when we knocked down PAF53-AID and quantified the levels of PAF49 after 3 h of IAA treatment, there was no significant change in PAF49 protein levels (Fig. 4, *C* and *D*). Next, we determined if PAF49 and PAF53 were degraded simultaneously. Was PAF49 bringing PAF53 to the proteasome, or was PAF53 targeted for degradation after PAF49? To test these two models, we treated cells with IAA, collected cells at multiple time points during the following 3 h, and performed Western blot analysis on the whole cell extracts to measure PAF49-AID and PAF53 levels (Fig. 4, *E* and *F*). The change with time was plotted with a nonlinear fit using GraphPad Prism. As indicated by the black dotted lines in Figure 4*F*, the half-life of PAF53 in these experiments is approximately two fold longer than the half-life of AID-PAF49. These results indicate that PAF53 is being degraded subsequent to the degradation of PAF49 instead of being degraded simultaneously with PAF49.

**Figure 5:**
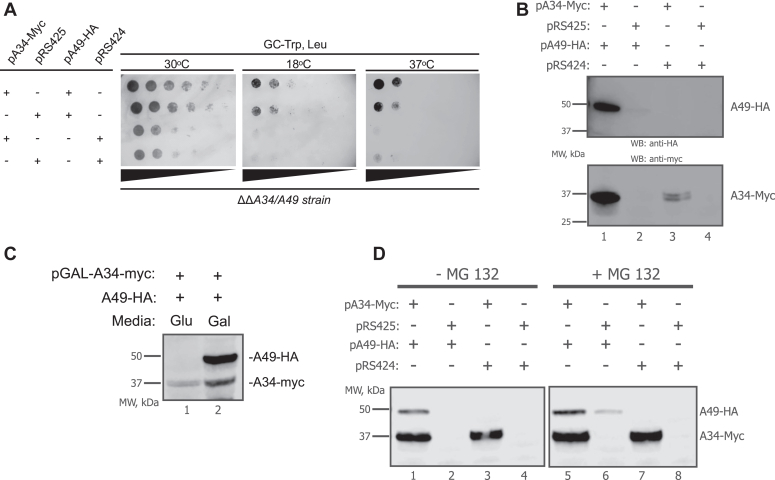
**Yeast A34 and A49 demonstrate co-dependent levels of expression.***A*, a double Δ*rpa34*/Δ*rpa49* deletion strain was transformed with the indicated plasmids that express either Myc-tagged wild type A34, HA-tagged wild type A49, and/or their corresponding empty vectors. Equivalent amounts of yeast cells were serially diluted and spotted onto glucose complete (GC) medium lacking leucine and tryptophan and then grown at the indicated temperatures. *B*, whole-cell extracts from the yeast strains in *A* were prepared from liquid cultures and then analyzed by Western blot using the indicated epitope tag antibodies. *C*, the double Δ*rpa34*/Δ*rpa49* deletion strain was co-transformed with a galactose inducible Myc-tagged A34 expression plasmid and an HA-tagged A49 expression vector. Cells were grown minimal media lacking Leucine and Tryptophan with glucose (GLU) as the carbon source or galactose (GAL). Whole extracts from these yeast strains were analyzed by Western blot using the indicated epitope tag antibodies. *D*, whole-cell extracts from the yeast strains in *A* were prepared from liquid cultures grown in the presence or absence of MG132 and then analyzed by Western blot. EV, empty vector.

Additionally, we determined whether yeast RPA49 and RPA34, the homologs to the mammalian heterodimer, are codependently expressed. Previous studies have reported that neither RPA34 and RPA49 are not essential for cell proliferation and viability (31, 32, 41, 48, 49). Specifically, the proliferation rate of RPA34^−/−^ cells is similar to wild-type. In contrast, KO of RPA49 shows a small growth defect at the permissive temperature, that is exacerbated at unfavorable growth temperatures. A similar growth defect is seen in the double RPA34^−/−^/49^−/−^ cells (Fig. 5*A*). The proliferation rate of cells ectopically expressing A49 was similar to those expressing both A34 and A49. Interestingly, when either RPA49 (Fig. 5*B*, *lane 2*) or RPA34 (*lane 3*) was ectopically expressed independently in the double KO background, they did not express well. However, when both were co-expressed, their levels significantly increased. This was also demonstrated in Figure 5*C*, where RPA49 did not accumulate until the expression of RPA34 was induced with the addition of galactose to the growth medium.

**Figure 6:**
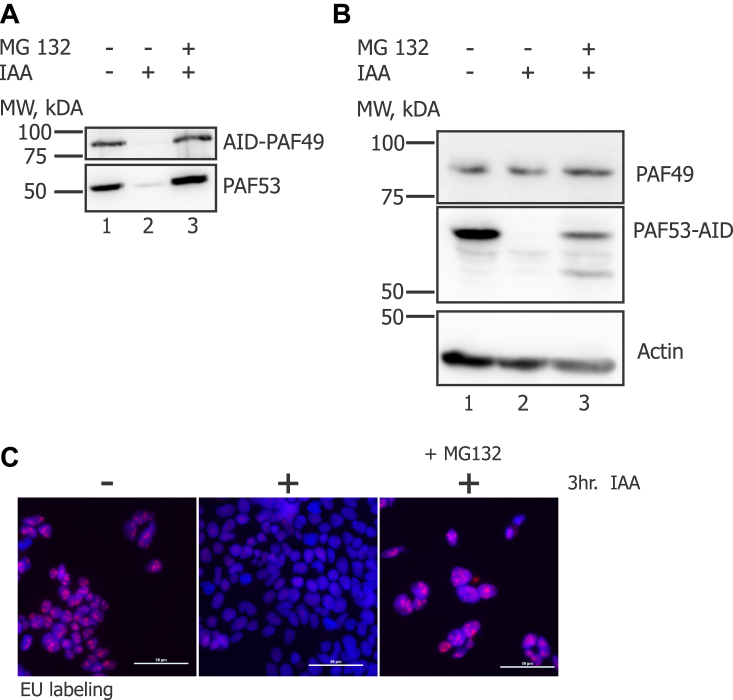
**Inhibition of the IAA-induced proteasomal degradation of either AID-PAF49 or PAF53-AID rescues rDNA transcription.***A*, cells with AID-PAF49 were treated + 1 mM IAA in the presence or absence of 10 mM MG132 for 3 h. Whole-cell extracts were prepared and analyzed *via* Western blot. *B*, the same experiment from *A* was performed with cells containing PAF53-AID. *C*, cells with AID-tagged PAF49 were treated 1 mM IAA or vehicle in the presence or absence of 10 mM MG132 for 3 h. Following the 3 h treatment, cells were pulsed with 5-EU for 15 min. *De novo* synthesized RNA was visualized as described (*red*) against a DAPI background (*blue*) (94). Scale bar = 50 μm.

TIR1 is an E3 ubiquitin ligase that recognizes auxin-inducible degrons bound to auxin and targets them for degradation *via* the proteasome (44, 45). We determined whether inhibiting the proteasome could rescue the degradation of PAF53 in PAF49-AID cells. Cells were treated with IAA in the presence or absence of 10 mM MG132, a proteasome inhibitor (62), and analyzed *via* Western analysis. As predicted, the addition of MG132 inhibited the degradation of AID-PAF49 and untagged PAF53 (Fig. 6*A*). We observed similar results when we performed the same experiment in PAF53-AID cells (Fig. 6*B*). Consistent with the observation that MG132 prevents the degradation of the heterodimer, we found that (Fig. 6*C*) treatment with MG132 in the presence of IAA could rescue rDNA transcription. Our results indicate that either ubiquitinated PAF49 is still able to function in Pol I transcription or there is a deubiquitinase in the nucleolus that is able to deubiquitinate PAF49 so that it can participate in rDNA transcription. Interestingly, we found that MG132 could partially stabilize RPA49 when it was ectopically expressed in the absence of RPA34 in yeast (Fig. 5*D*, lane 6). These results indicate that while yeast RPA34 is not required for rDNA transcription and cell proliferation, it plays an important role in stabilizing RPA49.

**Figure 7:**
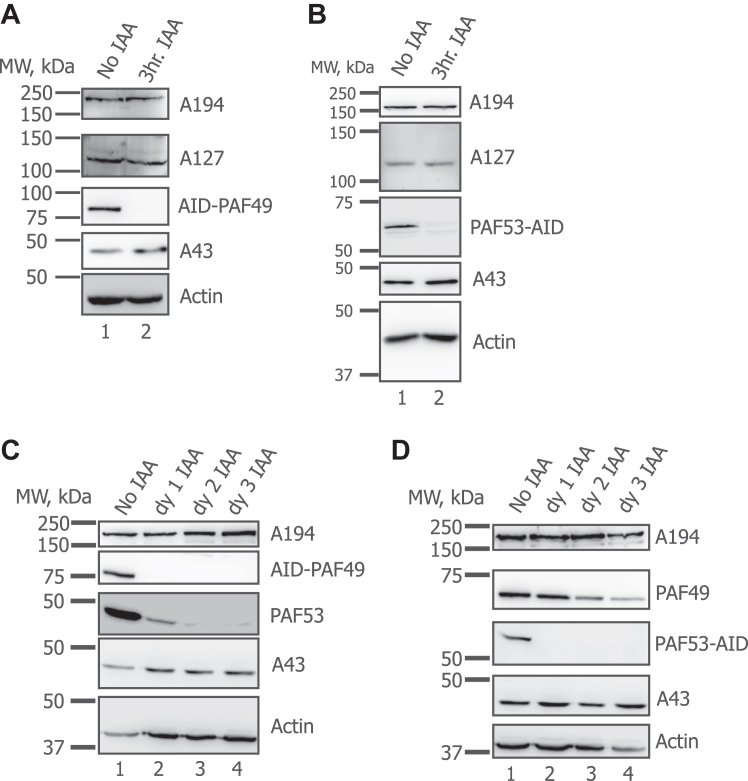
**Inhibition of rDNA transcription by depletion of PAF49 or PAF53 does not affect the steady-state levels of other Pol I subunits.***A*, cells expressing AID-PAF49 were treated with 1 mM IAA or vehicle for 3 h and were harvested and lysed in HEPES lysis buffer. The levels of multiple subunits of RNA Polymerase I (A194, A127, and A43) were determined by Western blot analysis. *B*, cells with PAF53-AID were treated with 1 mM IAA or vehicle for 3 h and were harvested and lysed in HEPES lysis buffer. The expression of the same Pol I subunits as in *A* were visualized by Western blot analysis. *C*, cells with AID-PAF49 were treated with IAA for 3 days. On each day, including day 0, cells were harvested and lysed as described previously. The expression of A194, PAF53, and A43 was determined by Western blot analysis. *D*, cells with PAF53-AID were treated with IAA for 3 days. On each day, including dy 0, cells were harvested and lysed as described previously. The expression of A194, PAF49, and A43 was determined by Western blot analysis.

Previous studies on BMH-21, a small molecule inhibitor of Pol I, have shown that RPA194 (POLR1A), the largest subunit of Pol I, is degraded within 3 h of treatment (63, 64, 65, 66). Therefore, we determined whether inhibiting Pol I transcription by targeting a Pol I subunit for degradation would also cause the degradation of other Pol I subunits. In Figure 7, panels A and B, we knocked down either PAF49 or PAF53 for 3 h and measured the levels of several other Pol I subunits by Western analysis. After 3 h of IAA treatment, we did not observe a decrease in the levels of three other Pol I subunits: RPA194, RPA127, and RPA43. These results corroborate the immunofluorescence data presented in Figure 3*F*. (The immunofluorescent signal for RPA194 did not disappear upon treatment with IAA.) We expanded this study by observing if prolonged knockdown of either PAF49 or PAF53 would lead to the degradation of Pol I subunits. Western blots demonstrated that treating AID-PAF49 cells or PAF53-AID cells with IAA for 3 days (Fig. 7, panels C and D, respectively) did not cause the degradation of either RPA194 or RPA43. Interestingly, the levels of PAF49 decreased slightly after PAF53-AID cells were treated with IAA for 2 days. However, the decrease was not nearly to the same extent that PAF53 was degraded (compare Fig. 7*D* with Fig. 4, *A* and *B*).

**Figure 8:**
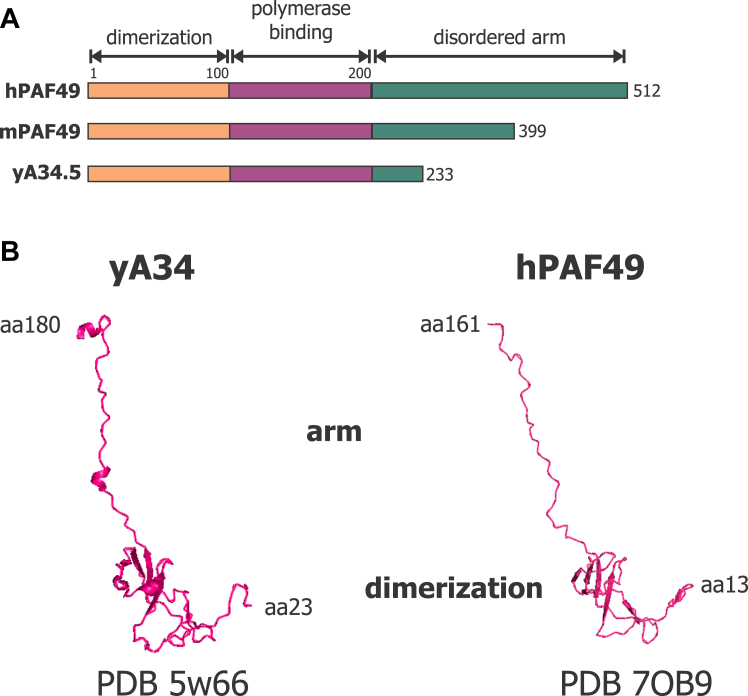
**Domains of yeast A34 and mammalian PAF49.***A*, cartoon depicting the modular domains of the yeast A34, human PAF49, and mouse PAF49 homologs. The domain names are labeled above with brackets. *Light orange* represents the dimerization domain, *magenta* represents the ordered arm that facilitates binding to the polymerase, and *green* represents the disordered arm of the PAF49 homologs. The domain boundaries of yA34 are adapted from (67). The demarcations for the domains of the mammalian PAF49 homologs are adapted from (30, 36, 61). *B*, the cryo-EM structures of yA34 (adapted from PDB 5w66) and hPAF49 (adapted from PDB 7OB9). The dimerization domain and the arm of the homologs are indicated. The amino acids (aa) represented in each structure are also shown.

As shown in Figure 8, the yeast RPA34 and its homologs consist of three main domains: the dimerization domain, an ordered arm, and a disordered carboxy-terminal region (67). The dimerization domain is responsible for dimerizing with RPA49 in yeast and PAF53 in mammals (61, 67, 68). The function of the ordered arm has not been investigated, but based on cryo-EM structures, we suspect that it is responsible for binding to the polymerase *via* interactions with A127, the second largest subunit (30, 68). Interestingly, the disordered arm of the mouse and human homologs is much larger than the yeast homolog (Fig. 7*A*). We found that (61) this segment is subject to post-translational modifications that regulate the association of PAF49 with the polymerase. When the sequences of yeast RPA34 and human PAF49 are compared, there is only approximately 18% identity between the two (69). But when the cryo-EM structures are compared (Fig. 8*B*), the structures are similar (30, 61, 67, 68, 70). We have also observed that the disordered arm of either homolog has not been resolved in any cryo-EM. This is most likely due to the intrinsically disordered nature of the arm.

**Figure 9:**
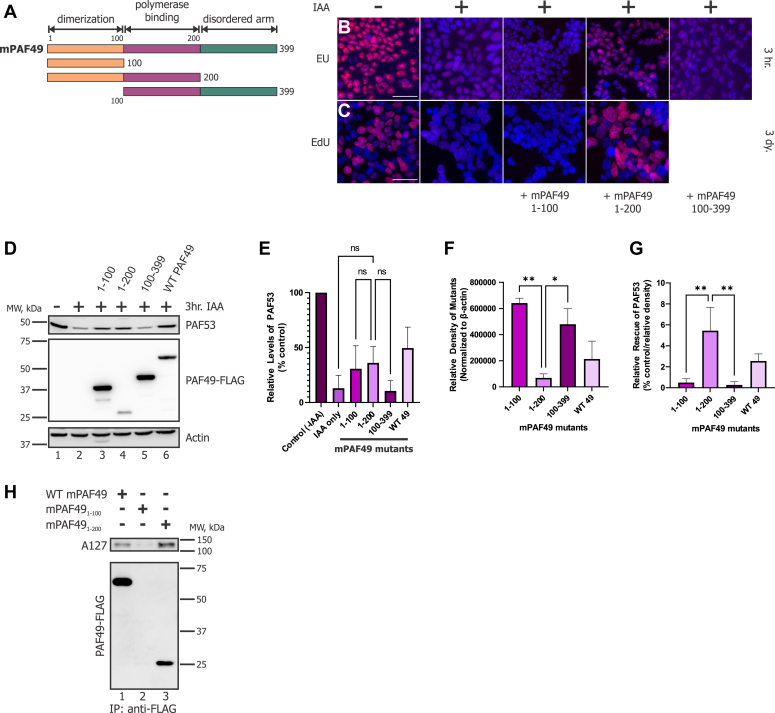
**The functionality of PAF49 requires the dimerization domain and adjacent sequences.***A*, diagrams of the mPAF49 constructs used in the following experiments. *B*, 24 h prior to IAA treatment, cells were transfected with the mPAF49 mutants indicated in (*A*). Cells were then treated with 1 mM IAA or vehicle for 3 h to knock down endogenous PAF49-AID. After 3 h, the cells were pulsed with 5-EU for 15 min and *de novo* synthesized RNA was visualized as described (*red*) against a DAPI background (94). Scale bar = 50 μm. *C*, to assess cell proliferation *via* active DNA replication, cells were treated with 1 mM IAA or vehicle for 3 days instead of 3 h as done in (*B*). Cells were then pulsed with EdU for 1 h and newly synthesized DNA was visualized as described (*red*) against a DAPI background. Scale bar = 50 μm. *D*, cells were transfected with either WT mPAF49 or one of the mPAF49 mutants described in *A* 24 h prior to IAA treatment. After 3 h of 1 mM IAA treatment, cells were harvested and lysed as described. The levels of hPAF53 and the mPAF49 mutants were determined *via* Western blot analysis. All mPAF49 constructs were tagged with the FLAG epitope. mPAF49_1-100_ is also tagged with the GST epitope in order to make the protein large enough to detect on a Western blot. This explains why mPAF49_1-100_ has a larger molecular weight than mPAF49_1-200_. *E*, three independent repeats of the WB data presented in (*D*) were performed. The relative levels of PAF53 were quantified and corrected with β-actin. The data were analyzed by one-way ANOVA with Tukey’s multiple comparisons test; ns indicates *p* > 0.05. *F*, for the WB data presented in (*D*), the relative expression levels of the ectopically expressed mPAF49 mutants (and WT). They were quantitated and normalized to β-actin. Significance was determined *via* a one-way ANOVA with Tukey’s multiple comparisons test. The error bars represent mean ± SD; ∗ = 0.05 to 0.01 and ∗∗ <0.01. *G*, to assess how well each mPAF49 mutant was able to rescue the level of PAF53 in the absence of endogenous PAF49, relative rescue of PAF53 was calculated for each construct (% control/relative density ∗ 1E4). This calculation resulted in an integer that could be used to compare the activity of each mutant. Significance was determined *via* a one-way ANOVA with Tukey’s multiple comparisons test. The error bars represent mean ± S.D; ∗ = 0.05 to 0.01 and ∗∗ <0.01. *H*, HEK293 cells were transfected with the indicated FLAG-PAF49 mutant. 48 h later, the cells were lysed, and the FLAG-tagged proteins recovered with immobilized anti-FLAG antibodies and analyzed by western blotting for FLAG and A127. PAF49 1 to 100 did not blot well (lane 2).

Next, we sought to determine which domains of PAF49 were essential for rDNA transcription and DNA synthesis. Figure 9*A* displays the modular domains of the mPAF49 mutants that were utilized for the experiments performed for this figure. We used EU labeling of newly synthesized RNA to determine which mutants were able to rescue rDNA transcription. Our results (Fig. 9*B*) demonstrate that of the constructs tested, only ectopically expressed mPAF49_1-200_ was able to support rDNA transcription. Similarly, when we used EdU labeling of newly synthesized DNA to assess whether cells are actively progressing through the cell cycle, we found (Fig. 9*C*) that only mPAF49_1-200_ is able to rescue cell proliferation. Together, these results indicate that both dimerization with PAF53 and binding to the polymerase are required for PAF49 to support rDNA transcription and cell proliferation. Additionally, the C-terminus of PAF49 that is not resolved in the cryo-EM structures is not essential for function (Fig. 9*B*). Since PAF53 is degraded in the absence of PAF49 (Fig. 4*A*), we determined which domains of PAF49 are responsible for the stabilization of PAF53. Our first model, that the dimerization domain of PAF49 would be sufficient to stabilize PAF53, was disproven (Fig. 9, *D*–*G*). PAF49_1-100_, the dimerization domain, did not rescue the levels of PAF53 efficiently (Fig. 9, *D*–*G*). While the dimerization domain was not sufficient to stabilize PAF53, it was essential. Mouse PAF49_100-399_ was unable to prevent PAF53 from being degraded (Fig. 9, *D*–*G*). Similar to the results from Figure 9, *B* and *C*, mPAF49_1-200_ was able to efficiently prevent PAF53 from being degraded in the absence of endogenous PAF49. Therefore, dimerization and binding to the polymerase are both required to stabilize PAF53. Cryo-EM structures have suggested that the ordered arm of PAF49 is required to facilitate binding to the polymerase. As we had hypothesized, we found that mPAF49_1-200_ was able to bring down polymerase (A127) while mPAF49_1-100_ was unable to (Fig. 9*H*, *lanes 2 and 3*). These results support our conclusion that PAF49 needs to be able to dimerize with PAF53 and bind to polymerase in order to support rDNA transcription, cell proliferation, and keep PAF53 stable.

**Figure 10:**
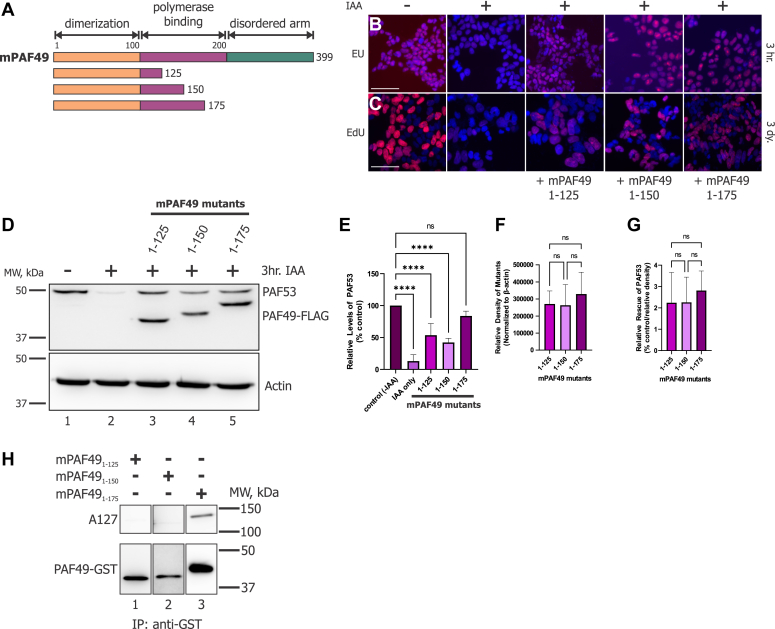
**Characterization of the “ordered arm” of PAF49: Identification of regions required for rRNA synthesis, PAF53 stabilization, and binding to Pol I.***A*, diagrams of the mPAF49 constructs used in the following experiments. *B*, 24 h prior to IAA treatment, cells were transfected with the mPAF49 mutants indicated in (*A*). Cells were then treated with 1 mM IAA or vehicle for 3 h to knock down endogenous PAF49-AID. After 3 h, the cells were pulsed with 5-EU for 15 min and *de novo* synthesized RNA was visualized as described (*red*) against a DAPI background. Scale bar = 50 μm. *C*, to assess cell proliferation *via* active DNA replication, cells were treated with 1 mM IAA or vehicle for 3 days instead of 3 h as done in (*B*). Cells were then pulsed with EdU for 1 h and newly synthesized DNA was visualized as described (*red*) against a DAPI background. Scale bar = 50 μm. *D*, cells were transfected with one of the mPAF49 mutants described in (*A*) 24 h prior to IAA treatment. After 3 h of 1 mM IAA treatment, cells were harvested and lysed as previously described. The levels of hPAF53 and the mPAF49 mutants were determined *via* Western blot analysis. All mPAF49 constructs were tagged with the FLAG epitope. *E*, three independent repeats of the WB data presented in (*D*) were performed. The relative levels of PAF53 were quantified and corrected with β-actin. *F*, for the WB data presented in (*D*), the relative expression levels of the ectopically expressed mPAF49 mutants were quantitated and normalized to β-actin. The error bars represent mean ± SD. *G*, to assess how well each mPAF49 mutant was able to rescue the level of PAF53 in the absence of endogenous PAF49, relative rescue of PAF53 was calculated for each construct (% control/relative density ∗ 1E4). This calculation resulted in an integer that could be used to compare the activity of each mutant. The error bars represent mean ± SD. *H*, HEK293 cells were transfected with the indicated GST-PAF49 mutants. 48 h later, the cells were lysed, and the GST-tagged proteins recovered with glutathione Sepharose and analyzed by western blotting for PAF49 and A127.

In order to further refine the function of the domain between aa100-aa200, mutants were constructed in 25aa increments from aa1-125 to aa1-175 of mPAF49 (Fig. 10*A*). In Figure 10, *B* and *C*, we utilized EU and EdU labeling to assess rDNA transcription and cell cycle progression, respectively. Each mutant was able to support rDNA transcription (Fig. 10*B*) and cell proliferation (Fig. 10*C*). To quantify this activity, we measured the ability of these different PAF49 mutants to rescue PAF53. As shown in Figure 10*E*, mPAF49_1-175_ was able to rescue the degradation of PAF53 most effectively, but when the activity of the mutants was corrected for their levels of expression, there were no significant differences between the activities of the three mutants (Fig. 10, *D*–*G*). To confirm that these mutants were in fact interacting with core Pol I, we determined if they would coimmunoprecipitate with A127. As shown in Figure 10*H*, only mPAF49_1-175_ was able to pull down polymerase. We thought the results were interesting since Figure 10, *B* and *C* demonstrates that all three mutants are able to support rDNA transcription and cell cycle progression. These data indicate that mPAF49_1-175_ is able to bind to polymerase more tightly than the other two mutants since its interaction with A127 stays intact after the *in vitro* immunoprecipitation experiment. Since there was no significant difference between the activity of the mutants tested in this figure, we decided to focus on the region between aa125-aa175 for our next set of experiments.

**Figure 11:**
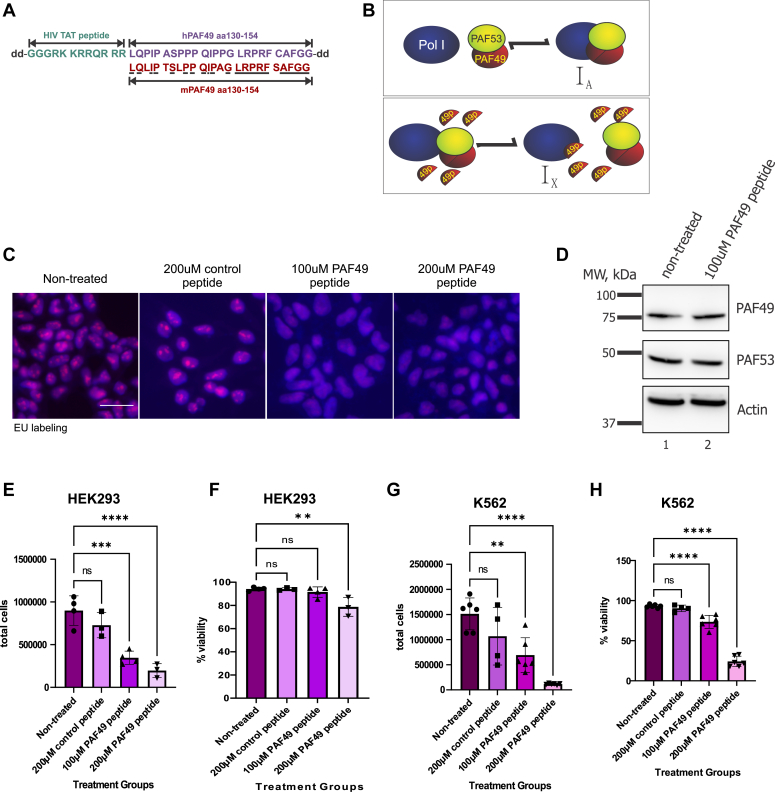
**Transduction of a peptide containing amino acids 130 to 154 of PAF49 can inhibit rRNA synthesis in cells.***A*, sequence of the hPAF49 peptide designed to inhibit rDNA transcription. The 2 d’s at the N- and C-terminal of the peptide represent d-alanine’s. The HIV TAT peptide sequence used to promote the transduction of the peptide into cells is in *green*. The hPAF49 sequence used to construct the peptide is in *purple* (aa130–154). A comparison of the mPAF49 sequence is in *red*. Identical amino acids between sequences are *underlined* in *black*. The peptide was provided from Peptide 2.0. *B*, the proposed mechanism of action of the PAF49 peptide. I_A_ represents active polymerase and I_X_ represents inactive polymerase. When the PAF49 peptide competitively binds to polymerase (A127), the heterodimer will no longer be able to associate with polymerase, leaving it inactive. *C*, HEK293 cells were treated with 100 to 200 μM of peptide or vehicle for 24 h. After 24 h, the cells were pulsed with 5-EU for 15 min and *de novo* synthesized RNA was visualized as described (*red*) against a DAPI background. Scale bar = 50 μm. *D*, HEK293 cells were treated ± 100 μM of peptide for 24 h and harvested as previously described. The levels of hPAF49 and hPAF53 were determined *via* Western blot analysis. *E*, HEK293 cells were treated with 100 to 200 μM of peptide or vehicle for 48 h. After 48 h, the cells were counted as described (79). Total cells/ml is displayed as mean ± SD. *F*, trypan blue exclusion was used to count live cells and measure percent viability within the cell population. Percent viability is displayed as mean ± SD. *G*, K562 cells were treated with 100 to 200 μM of peptide or vehicle for 48 h. After 48 h, the cells were counted as described (79). Significance was determined *via* a one-way ANOVA with Tukey’s multiple comparisons test. The error bars represent mean ± S.D; ∗∗ <0.01 and ∗∗∗∗<0.0001. *H*, trypan blue exclusion was used to count live cells and measure percentage viability within the cell population. Significance was determined *via* a one-way ANOVA with Tukey’s multiple comparison tests. The error bars represent mean ± *SD*; ∗∗∗∗<0.0001.

A marker of malignant cancer cells is pleiomorphic nucleoli that are associated with an increased rate of ribosome biogenesis (RB) (71). The rate-limiting step in RB is rDNA transcription by Pol I (53, 72, 73). Multiple studies have demonstrated that selectively inhibiting Pol I transcription in cancer cells causes cell death (57, 74, 75, 76, 77, 78). We hypothesized that a small peptide would disrupt the interaction between PAF49 and polymerase (A127), inhibit rDNA transcription, and induce cancer cell death. The experiments described above suggested that we focus on amino acids 125 to 175 of PAF49. When we aligned the mPAF49 and hPAF49 amino acid sequences between 125 and 175, we found that aa130 to 154 demonstrated the best identity (Fig. 11*A*). Based on our previous studies of the interaction of RRN3 with RPA43 (79), we sought to determine if that peptide would inhibit rDNA transcription *in vivo*. To facilitate the transduction of the peptide, we fused the hPAF49 peptide to a cell-transducing peptide based on the HIV TAT protein transduction domain (80). To inhibit cleavage of the peptide by proteases and peptidases, we attached two d-alanine’s to the N- and C-termini of the peptide (81). These are denoted as **d** in Figure 11*A*.

Our goal with the PAF49 peptide was to identify an essential Pol I protein–protein interaction that might be a novel therapeutic target to selectively treat cancer cells. We hypothesize that such a peptide would be able to compete for binding to A127 with the endogenous PAF49 protein (Fig. 11*B*). In this model, the binding of the peptide to A127 would compete with PAF49. Since we have determined that PAF49 is required for rDNA transcription (Fig. 2*A*), loss of PAF49 from Pol I would lead to the termination of Pol I transcription and cause either cell cycle arrest in normal cells or cell death in cancerous cells. First, we tested whether treatment with the PAF49 peptide could inhibit rDNA transcription in HEK293 cells. In Figure 11*C*, we demonstrate that treatment of HEK293 cells with the PAF49 peptide for 24 h caused the inhibition of rDNA transcription. Additionally, treatment of cells with the maximum concentration (200 μM) with a TAT-control peptide (79) for 24 h did not inhibit rDNA transcription (Fig. 11*C*).

Because PAF53 is rapidly degraded when PAF49 is knocked down, we determined whether dissociating the intact heterodimer from the polymerase would trigger the degradation of PAF53 and/or PAF49. To test this, we treated HEK293 cells with or without 100 μM of the PAF49 peptide for 24 h and visualized the protein levels of PAF49 and PAF53 *via* Western blot analysis. There was no change in the levels of PAF53 and PAF49 after peptide treatment (Fig. 11*D*).

Next, we confirmed our results from Figure 11*C* and determined whether the PAF49 peptide would be able to inhibit cell proliferation in non-transformed cells without being toxic. Treatment of HEK293 cells with 100 μM and 200 μM of the PAF49 peptide for 48 h caused an inhibition of cell proliferation (Fig. 11*E*). Alternatively, treatment of HEK293 cells with 200 μM of a TAT-control peptide caused no significant decrease in cell number. Additionally, there was no significant difference between the percent viability of control, TAT-control peptide, and 100 μM PAF49 peptide-treated cells after 48 h (Fig. 11*F*). Having established that the PAF49 peptide inhibited cell proliferation without being toxic to non-transformed cells, we tested if the PAF49 peptide could be used to induce cancer cell death. To this end, we treated K562 cells, a human chronic myelogenous leukemia cell line, with either 200 μM TAT-control peptide or 100 to 200 μM PAF49 peptide and measured cell number and percent viable cells *via* a trypan blue exclusion assay (Fig. 11, *G* and *H*). The TAT-control peptide did not inhibit cell proliferation or cause cell death. On the other hand, the PAF49 peptide caused a dose-dependent decrease in total cell counts (Fig. 11*G*) and an increase in cell death (Fig. 11*H*).

## Discussion

We have demonstrated that mammalian PAF49 is required to support rDNA transcription by RNA Pol I and cell proliferation, but it is not required for short-term cell viability (Fig. 2). These results conflict with studies performed in yeast that report RPA34, the homolog to PAF49, is not essential for proliferation or rDNA transcription (31, 32, 48, 49). Multiple studies from our laboratory and others have demonstrated that although there are similarities between the yeast and the mammalian Pol I systems (30, 41, 42, 69), there are also many differences that warrant the need to study this system in mammals in order to effectively target the process of rDNA transcription in disease models where ribosome biogenesis and/or Pol I transcription is dysregulated.

The observation that knock down of PAF49 resulted in cell cycle arrest, suggested that we would see arrest at either the G_1_/S or G_2_/M boundaries. However, we saw a statistically significant increase in the accumulation of cells in the S phase and a significant decrease in cells in both G_1_ and G_2_ (Fig. 3). This is in contrast to previous studies that have looked at cell cycle arrest when rDNA transcription was inhibited (82, 83, 84, 85). Those investigations reported cell cycle arrest at the G_2_/M boundary or in G_1_. We hypothesize that our results may differ from those previously reported due to the method of targeting Pol I. The previous studies referenced utilized either RNAi technology that knocked down a component of the Pol I apparatus or a small molecule inhibitor to target Pol I and inhibit rDNA transcription. Alternatively, using the Tir1-AID system allowed us to directly target and inhibit Pol I transcription within 3 h (Fig. 2). This method prevents the potential issue of cell compensation or off-target effects that can influence the results of the FACS analysis. Other methods such as RNAi technology takes multiple days to achieve knockdown of the protein of interest and the small molecule inhibitors used, such as actinomycin D, BMH-21, and CX-5461, have other targets besides Pol I. Interestingly, the results reported above (Fig. 3) are similar to those reported by our lab when PAF53 was knocked down *via* the same Tir1-AID system (41). We have two hypotheses to explain our cell cycle analysis results: (1) the cells are proceeding through the cell cycle at a greatly reduced rate that cannot be detected by the methods we used or (2) all of the cell cycle checkpoints are firing at once, causing the cells to arrest at whichever phase they are at in the cell cycle. Further examination is required to understand how cells arrest when Pol I transcription is rapidly and specifically targeted.

In addition to cell cycle arrest, we observed a significant increase in p53 accumulation over 6 days of PAF49 knockdown (Fig. 3). This is consistent with previous studies that have reported that inhibition of rDNA transcription can cause a p53-dependent nucleolar stress response that can result in cell cycle arrest or cell death depending on the physiology of the cell (52, 59, 86). Another hallmark of nucleolar stress is the reorganization of the nucleolus into a ring-like structure with caps (58, 87). As shown in Figure 3, our observations are consistent with previous studies that investigated the nucleolar stress response and its effects on nucleolar organization. Interestingly, while UBF, a general transcription factor of Pol I, reorganized into caps along the periphery of the nucleolus, A194, a subunit of Pol I, dispersed throughout the nucleoplasm instead of reorganizing with other components of the nucleolus. Additional studies need to be performed to determine if the core polymerase stays intact during the nucleolar stress response or if it breaks apart causing each core subunit to either reorganize into caps or disperse through the nucleoplasm independently.

Studies of cells treated with BMH-21, a small molecule inhibitor of Pol I transcription, has observed both A194 and A127 reorganizing into caps that co-localize with UBF upon treatment with BMH-21 and MG132 (to inhibit degradation of A194) (64, 66). Additionally, they have reported that targeting Pol I with BMH-21 causes the degradation of A194 and the partial degradation of A127 (64, 66). These observations differ from those reported above in Figures 3 and 7. We do not see a decrease in Pol I subunit levels when rDNA transcription is inhibited in response to the knockdown of either PAF49 or PAF53 (Fig. 7). Further, A194 dispersed into the nucleoplasm and did not reorganize into caps (Fig. 3). We hypothesize that the discrepancies between our results and the studies with BMH-21 could be due to targeting different subunits of the polymerase. In our study, we target two polymerase-associated factors that can be dissociated from the core polymerase. On the other hand, BMH-21 targets a subunit of the core polymerase. The effects of targeting a peripheral subunit *versus* a core subunit could differ.

As previously mentioned, PAF49 and PAF53 act as a heterodimer and are required to support Pol I transcription and cell proliferation (36, 41, 42, 61, 67). Our study is the first to report that PAF49 is required to stabilize the levels of PAF53. Interestingly, the vice versa response is not observed, that is, the knockdown of PAF53 does not lead to the rapid destabilization of PAF49. Studies performed with yeast demonstrated that RPA34 may play an important role in stabilizing RPA49, the yeast homolog of PAF53, although neither protein is essential. Based on the results displayed in Figure 5, deletion of RPA49 results in a slow growth phenotype, while deletion of RPA34 shows no observable change in growth compared to wild-type yeast. While there is no change in growth in the absence of RPA34, RPA49 levels are decreased in the absence of its dimerization partner. Further, treatment of RPA34 null yeast with MG132, a proteasome inhibitor, was able to partially rescue levels of RPA49 (Fig. 5). Since the cell wall of yeast is less permeable to MG132 than the cell membrane of mammalian cells (88), we would not expect RPA49 levels to be fully rescued. The results generated from the experiments performed with yeast have left us with multiple questions that require additional studies to answer: (1) if RPA49 does not express well without RPA34, how is there no growth defect when RPA34 is deleted? (2) how much RPA49 protein is required to support rDNA transcription by Pol I? (3) is full-length RPA34 required to stabilize RPA49? And (4) since our results demonstrate that wild-type levels of RPA49 are not required to support Pol I transcription, does RPA49 have another role in the nucleolus/nucleus? While the experimental procedures used to study the expression levels of the heterodimer in yeast do not directly mirror the experiments performed with mammalian cells, the results from the yeast studies suggest that yeast RPA34 like its human counterpart contributes to the stabilization of RPA49.

There are several models to account for the observation that the depletion of PAF49 caused the rapid degradation of PAF53. The two main hypotheses were: (1) when PAF49 was targeted for degradation *via* the Tir1-AID system, PAF53 was being brought to the proteasome alongside PAF49, (2) the degradation of PAF49 destabilized PAF53 and induced its subsequent degradation. We determined that when cells containing AID-PAF49 were treated with IAA, the half-life of PAF53 was approximately 2-fold longer than that of AID-PAF49 (Fig. 4). This indicates that PAF53 is targeted for degradation in response to PAF49 being rapidly degraded. Additionally, we found that when PAF49 levels were reduced *via* treatment with siRNAs to PAF49, PAF53 levels were also reduced (data not shown). The observation that the two proteins demonstrate a co-dependent regulation in mammalian cells is consistent with our earlier observations that in 3T6 cells the levels of both proteins are reduced by 70% in response to serum starvation (36). Further studies need to be performed to determine how PAF53 is being targeted for degradation in the absence of PAF49.

As previously stated, RPA34, the yeast homolog of PAF49, is not essential to support rDNA transcription and wild-type levels of cell proliferation. Since it has been determined to be non-essential, no studies have been performed to determine how RPA34 functions during Pol I transcription. Additionally, there have been no studies to determine the roles PAF49 may play during rDNA transcription. To this end, we determined which domains of PAF49 were sufficient and/or essential to support rDNA transcription, cell proliferation, and rescue the levels of PAF53 when endogenous PAF49 is knocked down. The RPA34 family of proteins contains three domains: a dimerization domain, an ordered arm, and a disordered C-terminal tail (Fig. 8). The dimerization domain is responsible for facilitating heterodimerization with RPA49 in yeast and PAF53 in mammals (61, 67). Based on cryo-EM structures of both the yeast and the mammalian Pol I structures, we hypothesized that the ordered arm facilitates binding between the heterodimer and core polymerase (30, 43, 68). The results shown in Figure 9 confirm this hypothesis: The dimerization domain of PAF49 (aa 1–100) is insufficient to support binding to the polymerase, but the addition of the ordered arm (aa 100–200) is sufficient to facilitate binding to Pol I. One study from our laboratory has demonstrated that the disordered C-terminal tail of PAF49 contains sites for post-translational modifications that regulate its binding to polymerase (36). Our data indicate that the dimerization domain of mPAF49 (aa 1–100) is insufficient to support rDNA transcription and cell proliferation (Fig. 9). Interestingly, it is also insufficient to rescue PAF53 stabilization in the absence of endogenous PAF49. We had originally hypothesized that dimerization alone would be able to stabilize PAF53, but based on our results shown in Figure 9, the dimerization domain and the ordered arm of mPAF49 (aa 1–200) are necessary to stabilize PAF53 and support rDNA transcription and cell proliferation. Additionally, mPAF49 without the dimerization domain (aa 100–399) is unable to support rDNA transcription and rescue PAF53 stabilization (Fig. 9).

**Figure 12:**
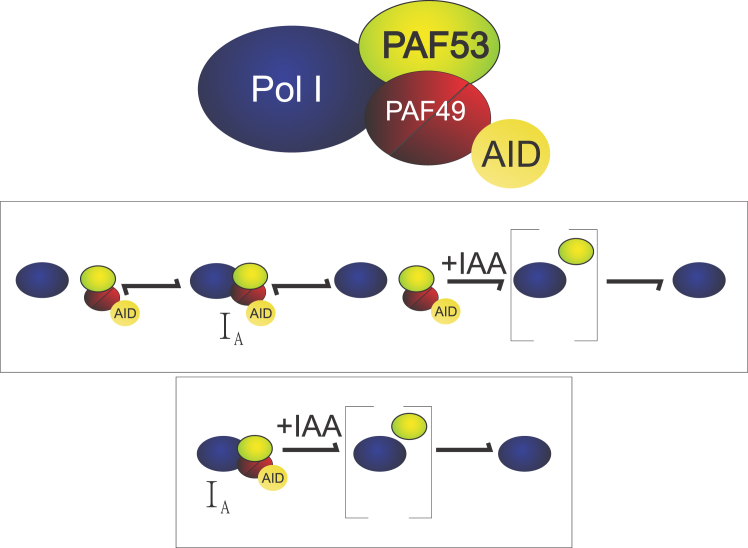
**Working model for the role of PAF49 in rDNA transcription.** PAF49 plays an important role in anchoring the heterodimer to the core polymerase. Without PAF49, the interaction between PAF53 and Pol I is destabilized causing PAF53 to disassociate from the polymerase. Pol I without the heterodimer is inactive. I_A_ represents active polymerase.

Our current working model, depicted in Figure 12, shows that under normal conditions, PAF49 and PAF53 interact as a heterodimer and can associate and dissociate from Pol I in equilibrium, but the transcriptionally competent form of Pol I must include the heterodimer (Fig. 12, I_A_). When IAA is added to rapidly knock down PAF49, PAF53 rapidly dissociates from the core polymerase and is degraded, resulting in a form of Pol I that cannot transcribe rDNA. Our model indicates that PAF49 plays an important role in anchoring the heterodimer to the polymerase. If PAF49 is unable to bind to polymerase and dimerize with PAF53, PAF53 is unable to form a stable interaction with Pol I, causing it to be targeted for degradation. The results from the experiments performed in yeast (Fig. 5) also point to RPA34 (PAF49 homolog) playing an important role in stabilizing RPA49’s (PAF53 homolog) interaction with Pol I, since the knockout of RPA34 causes a decrease in the expression of RPA49. A previous study also demonstrates that the absence of RPA34 results in a less stable interaction between RPA49 and the polymerase (32).

We utilized our working model to construct a small hPAF49 peptide that would be used to disrupt the interaction between A127 and endogenous PAF49 (Fig. 11). If our model is correct, the PAF49 peptide would competitively bind to the polymerase, causing the heterodimer to disassociate from Pol I causing it to become an inactive complex. When HEK293 cells were treated with the PAF49 peptide, rDNA transcription and cell proliferation were inhibited. The PAF49 peptide had a small effect on cell viability at 200 μM. Since many studies have demonstrated that inhibiting rRNA synthesis in cancer cells causes cell death (64, 76, 79, 89), we treated a cancer cell line, K562 cells, with the peptide and observed a dose-dependent decrease in the number of viable cells (Fig. 11). These results indicate that the interaction between PAF49 and the core Pol I (A127) can be used as a chemotherapeutic target. Our studies have also identified other possible drug targets, such as the interaction between PAF49 and PAF53, that could be utilized in cancer therapy.

Interestingly, treatment of HEK293 cells with the hPAF49 peptide did not trigger the degradation of PAF53 or PAF49 (Fig. 11). Based on data presented in this paper and data presented in previous papers from our laboratory, we had developed two hypotheses: (1) dissociating the heterodimer from the polymerase would target PAF53/49 for degradation or (2) dissociating an intact, functional heterodimer from the polymerase would not cause PAF53/49 to be targeted for degradation. Previous data from our lab suggests that stabilization of PAF49/PAF53 only requires their coexpression (61). We have found that the simultaneous ectopic expression of the two proteins results in their stabilization, even when their levels exceed those of core Pol I. Further, our laboratory has also demonstrated that serum-starving NIH3T3 cells cause the heterodimer to dissociate from the polymerase without causing any changes in their protein levels or disrupting the ability for PAF53 and PAF49 to heterodimerize (36). Overall, the results from Figures 9 and 11, and other studies from our lab demonstrate that the stabilization of PAF53 requires the protein expression of an active form of PAF49.

Further studies need to be performed to understand how PAF49 functions during transcription once it anchors the heterodimer to Pol I. Does PAF49 have a function outside of facilitating the interaction between the polymerase and the heterodimer? Further, what role does the disordered C-terminal tail play during rDNA transcription? Our data (Fig. 9) have demonstrated that the C-terminal tail of PAF49 is not required to support Pol I transcription. Does the tail only serve as a way to regulate rDNA transcription by regulating the association of the heterodimer with the core polymerase?

## Experimental procedures

### Cell culture, transfection, selection, and analysis

HEK293 cells (ATCC) were cultured in Dulbecco’s modified Eagle’s medium (Corning) containing 10% fetal bovine serum (ATLANTA biologicals) and antibiotic/antimycotic (Invitrogen). The human B-lymphoma cell line (K562) used in this study was provided by Dr Carol Webb (OMRF) and was characterized previously (90). K562 cells are grown in suspension in RPMI1640 media (Gibco) supplemented with 10% heat-inactivated FBS, 1 mM pyruvate, 50 μM beta-mercaptoethanol, 25 mM HEPES, and 1% Penn/Strep (Invitrogen). All cells were routinely passed at 1:10 dilution every fifth day and were not used after the 20th passage. In some experiments, transfection of HEK293 cells was carried out as described previously (36, 79) using polyethyleneimine (91) or TransIT-LT1 transfection reagent (92). We followed the transfection protocol recommended by the supplier. When PEI was used for transfection, the medium was changed after 6 h to remove the PEI. To select for HEK293 cells with the recombinant insert (GFP-Puro^R^-AID), puromycin (0.4 μg/ml) was added to the medium, and viable cells were cloned *via* limiting dilution. Cell counts, trypan blue exclusion, and cell cycle analysis were carried out as described (79, 93).

### Click chemistry to visualize RNA transcription and DNA synthesis

Cells were seeded on borosilicate glass coverslips that were coated with poly-L lysine (Millipore Sigma). To determine whether specific constructs rescued rDNA transcription or cell proliferation, the cells were transfected with a vector expressing the PAF49 construct to be tested. Twenty-four hours later, 1 mM IAA (IAA (Sigma-Aldrich) was made fresh by dissolving in water immediately before use) was added to the culture medium for 3 h (when visualizing RNA transcription) or 3 days (when visualizing DNA synthesis. After the indicated time, all cells were pulsed with either 1 mM 5-ethynyl uridine (100 mM EU stock solution) in sterile water, (Click Chemistry Tools); for RNA labeling for 15 min or 10 μM ethynyl deoxyuridine (10 mM EdU in DMSO, Click Chemistry Tools) for DNA labeling for 1 h. Cells were then fixed with 4% paraformaldehyde in 1X PBS for 15 min at room temperature (RT). Following fixation, they were permeabilized and blocked with 5% BSA and 0.5% Triton X-100 in 1X PBS for 20 min at RT. After permeabilization, cells were washed in 3% BSA in 1X PBS for 5 min and processed directly for the click-iT reaction. The click-iT mixture contained 1X PBS, 4 mM CuSO_4_ (Sigma-Aldrich), 5 μM AF594 azide (Click Chemistry Tools), and 40 mM sodium ascorbate (Sigma-Aldrich). The reaction was incubated for 1 h at RT in the dark. This protocol was adapted from a protocol previously described (94). This was followed by three washes with 3% BSA in 1X PBS for 5 min each. The coverslips were additionally washed three times with sterile water for 10 min each. The coverslips were then mounted with Prolong Diamond Antifade Mountant with DAPI (Invitrogen) used as recommended by the supplier. All cells on coverslips were imaged with an epifluorescent microscope at 40× magnification.

### Immunocytochemistry

The cells were fixed with 4% (weight/volume) paraformaldehyde in PBS for 15 min, washed with PBS, solubilized in 0.5% (volume/volume) Triton X-100 (Sigma) in PBS for 10 min and washed again before being incubated in primary antibodies that included: fibrillarin (Sigma, AnA-N), rabbit POLRIE/PAF53 antibody at 1:100 (Proteintech, 16145-1-AP), anti-UBF (provided by Edward Chen at the University of Florida) at 1:600. Incubations were for 1 h before cells were washed, and then finally incubated in anti-human, anti-mouse, or anti-rabbit Alexa-fluor secondary antibodies at 1:200 (Invitrogen, A-11013, A-11014, A-11001, A-11005) for 1 h. The cells were then mounted using Vectashield antifade mounting media with DAPI (Vector Laboratories Inc, H-1200). Immunolabeled cells were imaged using a Nikon Eclipse Ti fluorescence microscope with 60X objective and 1.4NA and 4 channels of fluorescence.

### Yeast growth assays

The genetic background for the double Δrpa34/Δrpa49 deletion strain is as follows: MATa ade2-1 ura3-1 trp1-1 leu2-3112 his3-11,15 can1-1, rpa49Δ::His3MX6, rpa34Δ::HphB. This strain was transformed with high copy 2 micro yeast expression vectors in their empty vector forms or variants that express C-terminally Myc-tagged A34 (pRS425-A34-Myc) or C-terminally HA-tagged A49 from their native promoter and termination sequences. Cells were grown in minimal glucose-containing media lacking Leucine and Tryptophan (GC-Leu,Trp). Spotting assays were performed on glucose complete plates lacking leucine and tryptophan. Equivalent amounts of cells were pelleted and washed with sterile deionized water. Cells were pelleted again and resuspended in sterile deionized water, serially diluted using five fold serial deletions, and then 5 μl of the diluted cells were spotted on the plates and grown at 30 °C, 18 °C, and 37 °C. Plates were grown the 2 to 3 days and imaged with a LiCOR Odyssey imager. For proteasome inhibitor treatments, cells treated with MG132 were grown in the presence of 75 μM MG132 (Selleckchem S2619) for 3 h at 30 °C. For galactose induction, cells were first grown in minimal glucose-containing media lacking leucine and tryptophan and then equivalent amounts of cells were used to inoculate galactose-containing media lacking leucine and tryptophan. C-terminal Myc-tagged A34 was under the control of the galactose-inducible GAL10 promoter (pPGAL10-A34-Myc).

### Yeast protein expression levels

Whole-cell extracts were prepared as described in (95) with minor modifications. In brief, ∼5 ml of yeast cells were grown in a glucose-complete minimal medium lacking leucine and tryptophan to an OD ∼1.0. Cells were washed with water, pelleted, and resuspended in 0.1 M NaOH for 5 min at room temperature. Cells were pelleted again and resuspended in 2× SDS sample buffer (Bio-Rad) and heated at 95 °C for 5 min. Cell debris was pelleted, and the soluble extract was collected and analyzed by SDS-PAGE and Western blotting. Proteins were resolved on 4 to 20% Tris-glycine polyacrylamide gradient gels (Bio-Rad) in 1× Tris-glycine SDS buffer, transferred to low-fluorescence polyvinylidene difluoride (Millipore), and probed with mouse anti-c-Myc (BioLegend, 9E10) and mouse anti-HA (Santa Cruz, F-7) antibodies.

### Constructs

The WT and deletion mutants of mouse PAF49 used in these experiments were described previously (36, 61) and are based on the NCBI Reference Sequence NP_665821.1. In these constructs, PCR was used to insert a FLAG-tag onto the N-terminus of the WT or mutant mouse PAF49 gene that was cloned into either pcDNA3.1 (Addgene) or pEBG-GST (Addgene) mammalian protein expression vectors.

### gRNA design

We used CHOPCHOP as described (96) to design a gRNA to target exon 1 of human PAF49. Oligos were synthesized by Sigma-Aldrich with a 20-bp gRNA sequence containing a 5′ overhang CACC and 3′ overhang CAAA to facilitate cloning into the Bbs1 site of pX330-YFG-PITCh vector (Addgene) (97). The set of gRNAs that we used to target exon 1 of human PAF49 were as follows: 5′ -TCAACCCGCACCCTCACCGC- 3′ and 5′ -GCGGTGAGGGTGCGGGTTGA- 3′. The oligonucleotides were annealed and ligated into the pX330-YFG-PITCh vector as previously described by Lin *et al.* (97). The construct was confirmed *via* sequencing.

### Changing microhomologies on the pN-PITCh-F-AID vector

The 20-bp microhomologies to direct MMEJ are based on the genomic sequences of human PAF49 adjacent to the gRNA cut site. Specifically, they were designed 3 bp before and after the PAM domain (Fig. 1*A*). Microhomologies on the pN-PITCh-F-AID vector were changed by PCR and cloned into the vector backbone with Gibson assembly. The 20 bp microhomology sequences used to knock in the AID tag at the correct locus on the human PAF49 gene were as follows: 5′ -GGAGGAGCCCCAGGC**A**GGCG- 3′ and 5′ -CCGTCAACCCGCACCCTCAC- 3′. The PAM sequence is underlined and a C to A mutation (bolded) was made to help prevent cleaving of the MMEJ repaired sequence by any remaining Cas9. To change the microhomology sequence in the pN-PITCh-F-AID vector, we followed what was described previously (97). For the Gibson assembly, NEBuilder HiFi DNA Assembly Master Mix (NEB) was used as recommended by the supplier.

### Microhomology-mediated end joining and clone selection

As described in Lin *et al.* (97), HEK293 cells were co-transfected with the px330 and pN-PITCh-F-AID vectors. The px330 vector expresses Cas9 and two guide RNAs (gRNAs), one targeting the first exon of the human PAF49 (hPAF49) gene and the other targeting the pN-PITCh vector to release the repair fragment containing the AID tag. The pN-PITCh vector that we used contains GFP, the puromycin resistance gene, and a FLAG-tagged AID sequence. Three days after transfection, cells were selected with 0.4 μg/ml puromycin and cloned by limiting dilution. After cloning, the success of recombination was confirmed by PCR, as described previously (47), using the forward and reverse primers, 5′ -ATGATGCCCTACCCCTTTGGATC- 3′ and 5′ -CAAGTCCTGCTTGCAGGTAC- 3′. The primers were constructed from the genomic sequence directly flanking the inserted cassette to ensure the recombination occurred at the correct position on the PAF49 gene. The PCR products were cloned in PCR blunt II TOPO (Invitrogen). Both strands of four separate clones were sequenced. The analysis demonstrated that the sequence of all four clones was identical.

### Ligand and Immunoaffinity purification

Forty-eight hours post-transfection, whole cell lysates were prepared as described previously (61). Cells were scraped in RIPA buffer (10 mM Tris-HCl, pH 7.5, 150 mM NaCl, 0.5 mM EDTA, 0.1% SDS, 1% Triton X-100, 1% deoxycholate, 2.5 mM MgCl_2_, containing 1 mM PMSF and Pierce protease inhibitors (Thermo Fischer)) and used immediately as described (35, 98, 99). The lysates were tumbled with either anti-FLAG M2 affinity gel (Sigma Aldrich) or Glutathione Sepharose 4B (GE Healthcare) for 2 h at 4 °C. The beads were boiled in 2X SDS sample buffer, and the eluted proteins were analyzed *via* SDS-PAGE and Western blotting.

### Immunoblotting

Cells used for Western blot analysis were harvested in HEPES lysis buffer (20 mM HEPES, pH 7.9, 0.5 mM EDTA, 2% SDS, containing 1 mM PMSF and Pierce protease inhibitors (Thermo Fischer)). Bio-Rad D-C protein assay kit was used to perform protein determinations on whole cell extracts with bovine serum albumin as the protein standard. Proteins were resolved on 12% SDS polyacrylamide gels and transferred to an Immobilon-P PVDF membrane (Millipore) as described (35). Proteins were detected with the following antibodies: rabbit anti-PAF49 (GeneTex, 1:1000), anti-β actin-HRP (Sigma, 1:6000), mouse anti-p53 (sc-126 Santa Cruz, 1:1000), rabbit anti-PAF53 (Proteintech, 1:1000), rabbit anti-PolR1a (Aviva, 1:1000), rabbit anti-PolR1b (AB Clonal, 1:2000), rabbit anti-RPA43 (Bethyl, 1:2000), and anti-FLAG M2-HRP (Sigma, 1:1000). Visualization was performed as described (61). Developed Western blots were scanned with a Chemi-Doc MP imaging system (Bio-Rad). Protein densitometry analysis was conducted using UVP software, and the mean value normalized with loading control was used as the final protein band quantification.

### Statistical analysis

All experiments were reproduced at least three times. Quantitative results that required comparisons between two groups were subject to statistical analysis using a two-tailed Student’s *t* test. To determine statistical significance between more than two groups, a one-way ANOVA was used followed by either Dunnett’s multiple comparison test or Tukey’s multiple comparison test. Interactions between multiple factors were tested with two-way ANOVA followed by Tukey’s multiple comparison test. Data met assumptions of all parametric tests performed (*i.e.*, normal distribution and similar variances). All statistical analyses were performed with GraphPad Prism. All data in bar graphs and XY plots are shown as mean ± SD of independent replicates in all figures with n ≥ 3.

## Data availability

All data are contained within the manuscript.

## Conflict of interest

The authors declare that they have no conflicts of interest with the contents of this article.
